# Underutilization of CRS, HIPEC & PIPAC in peritoneal mesothelioma across a German nationwide cohort

**DOI:** 10.1186/s12957-026-04209-6

**Published:** 2026-02-18

**Authors:** Veronika Müller, Matthias Hornung, Jens Werner, Hans Jürgen Schlitt, Miklos Acs

**Affiliations:** Department of Surgery, University Hospital Regensburg, Regensburg, Germany

## Abstract

This retrospective cohort study utilizes a nationwide database to provide information about the epidemiology and surgical treatment practices of peritoneal mesothelioma in Germany over the years 2019 to 2024.

Data collected by the German Institute for the Hospital Remuneration System (InEK) was examined by age, sex, treatment with HIPEC, PIPAC, or peritonectomy, ICU stay, type of hospital, and duration of stay. Additionally, the Patient Clinical Complexity Level (PCCL) - ranging from 0 to 6 - was analyzed.

In total 3394 patients with peritoneal mesothelioma were recorded during the years 2019 to 2024 in Germany, with an average annual incidence of 0.68 per 100,000 inhabitants. The mean duration of hospital stay was 9 days. The median PCCL was 1. The median patient age group was 65 to 74. 16% of patients had an ICU stay. Most patients were treated at maximum care hospitals with over 1000 beds. Only 9% of patients received HIPEC treatment. 15% received some form of peritonectomy. PIPAC was performed on only 5% of the patients. In total, considering that patients can be coded with multiple procedures, 20% of all patients received surgical therapy.

It was demonstrated that only a small percentage of patients received HIPEC, CRS, or PIPAC. This shows a grave discrepancy between guideline-recommendations and actual clinical practice. Potential reasons could be a lack of consideration of these procedures or misleading economic incentives. Ultimately, the findings highlight the need for increased awareness of peritoneal mesothelioma and underscore the importance to address the aforementioned observed discrepancy.

## Introduction

Peritoneal mesothelioma is a primary malignancy of the peritoneum with the disease derived from epithelial and mesenchymal cells [1]. With an annual incidence of 0.1/100,000 being reported in Germany, it is the most common cause of primary peritoneal surface malignancies [2]. The condition is categorized into borderline forms such as multicystic peritoneal mesothelioma, well-differentiated papillary peritoneal mesothelioma, or malignant forms like diffuse peritoneal mesothelioma [3]. Malignant mesothelioma is subdivided into three different histological subtypes: epithelioid, sarcomatoid, and biphasic [4–6]. Each subtype exhibits a unique prognosis with the most common type, the epithelioid version, having been recorded with the longest overall survival and the rare sarcomatoid versions being the most aggressive [2, 7].

Peritoneal mesothelioma poses a unique challenge in cancer treatment with patients often being diagnosed at a later stage of the disease with a vast spread across multiple surfaces and peritoneum. The therapy of peritoneal mesothelioma is further complicated by systemic chemotherapy not adequately affecting the peritoneum [8–10]. Instead, a surgical treatment consisting of cytoreductive surgery (CRS) accompanied by hyperthermic intraperitoneal chemotherapy (HIPEC) is the recommend treatment option [1, 3, 5, 7, 11, 12], with the longest overall survival [13]. This is stated in multiple international guidelines such as the 2023 NCCN [7] and 2022 PSOGI/EURACAN guidelines [12], as well as the 2023 consensus guidelines for peritoneal surface malignancies [1] and the Chicago consensus guidelines of 2020 [11].

Patients eligible for CRS and HIPEC are those with resectable disease burden, epithelioid or in some cases biphasic subtypes and enough fitness to endure the burden of surgery and HIPEC, classified by ECOG 0–1 [7]. Only patients that do not meet this criteria, and therefore do not qualify for the surgery, should be treated with solely systemic chemotherapy [1, 7]. However, these patients could also undergo pressurized intraperitoneal aerosol chemotherapy (PIPAC) [2]. If under these treatments patients do become resectable, they should be subjected to CRS and HIPEC secondarily [1]. At the presence of certain risk factors in patients who are eligible for CRS and HIPEC, such as lymph node or thoracic metastases, a PCI larger than 17, the biphasic subtype, or a Ki-67 over 9% a neoadjuvant [7, 14] and adjuvant chemotherapy is recommended [3].

In this study we utilize a nationwide database to provide information about the epidemiology of patients with peritoneal mesothelioma in Germany during the years 2019 to 2024. And examine the use of surgical therapy, especially of peritonectomy procedures in the framework of CRS and HIPEC, as well as the performance of PIPAC.

## Materials and methods

The German “Institut für das Entgeltsystem im Krankenhaus” (InEK), which translates to Institute for the Hospital Remuneration System, is a public data base. It contains anonymized data of the diagnostic codes assigned to patients and the subsequent treatments performed at various hospitals, as well as patient demographic data, information about hospital size, and duration of stay. It is utilized to analyze and adapt the financial resources appointed to hospitals. The diagnoses are listed by the assigned ICD-10 codes and performed therapies are noted by the “Operationen‑ und Prozedurenschlüssel” code (OPS), which stand for operations and procedures. Additionally, the system shows the patient clinical complexity level (PCCL), a score assigning a number between 0 (low) to 6 (high) to the level of severity of complications and comorbidities that arise within the selected patients.

We identified patients with peritoneal mesothelioma from the data by the ICD-10 code C45.1. We searched for the applied treatments utilizing the following OPS-codes:

‐ 8-546.0: HIPEC.
‐ 5-549.b: PIPAC.
‐ 5-543.4: Excision and dissection of peritoneal tissue aggregating the subcodes:
  - ○ 5-543.40: partial.
  - ○ 5-543.41: (sub)total.
  - ○ 5-543.42: local.

We used peritonectomy as a measure to detect CRS, as this is an essential part of the surgery [2], that has to be performed independently of the tumor invasion into other organs.

This data was then analyzed by cases per year, sex, length of hospital stay, age distribution, PCCL, types of hospitals treating these patients, and stays at the intensive care unit (ICU).

Data visualization and descriptive statical analysis was carried out utilizing MATLAB Version R2024b. Additionally, the NCI Joinpoint Regression Program, Version 5.4.0, was applied to examine if a trend in the overall incidence of peritoneal mesothelioma can be observed.

## Results

In total, 3394 cases of peritoneal mesothelioma were recorded in the years 2019 to 2024 in Germany. From that an average annual incidence of 0.68 per 100,000 inhabitants was calculated. Performing the Joinpoint Regression analysis of the incidence during the years 2019 to 2024 a significant decrease in incidence with an annual percent change (APC) of -5% could be detected (*p*-value 0.0032; CI -9% to -2%).

**Fig. 1:**
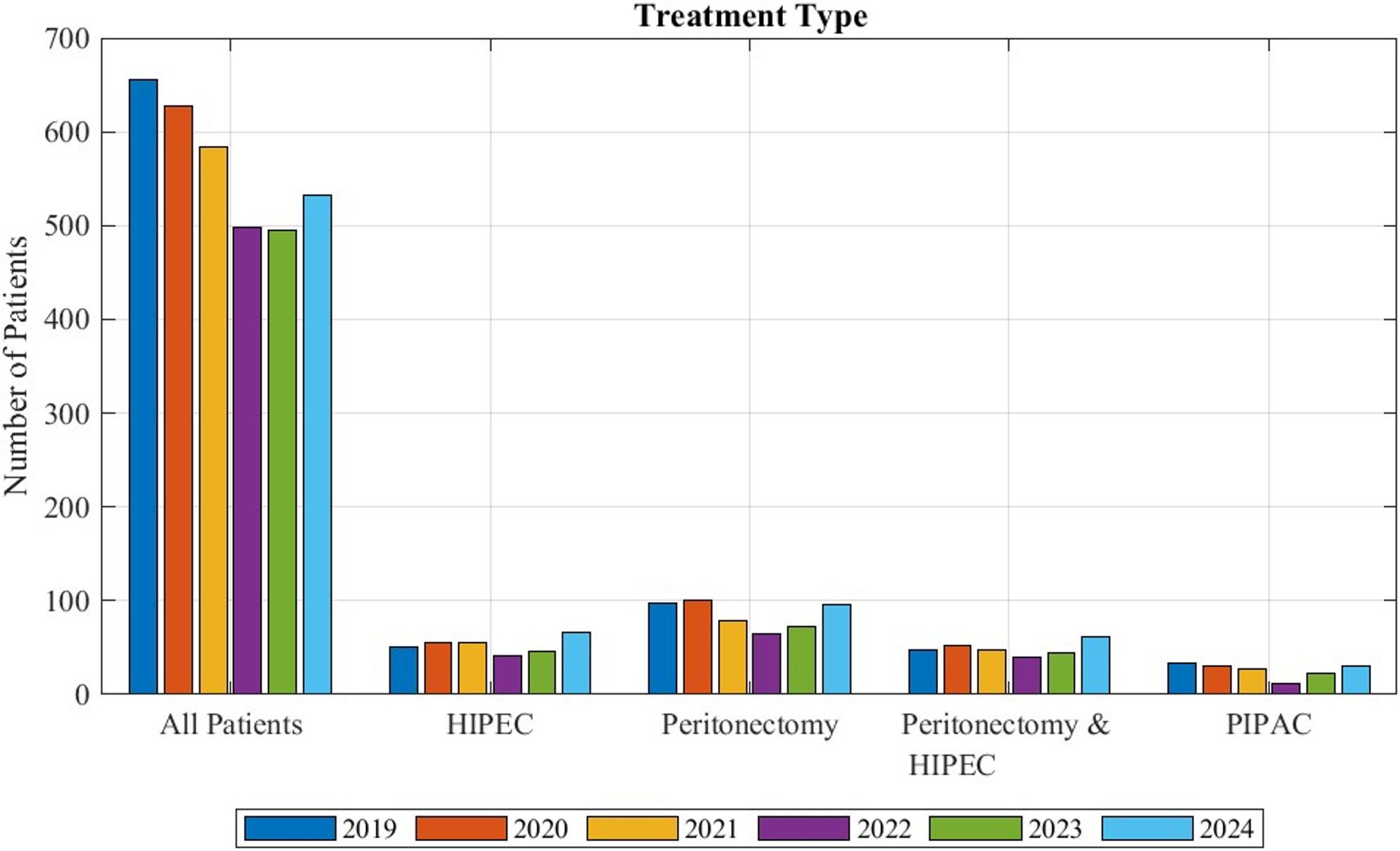
Patients with peritoneal mesothelioma coded with the examined procedures during the years 2019 to 2024 in Germany in relation to the total number of patients recorded with the diagnosis. “All patients” designates the total number of patients diagnosed with peritoneal mesothelioma. Peritonectomy & HIPEC refers to the patients for whom both procedures were coded simultaneously

During the recorded time frame HIPEC was performed on 318 patients, indicating that only 9% of patients with peritoneal mesothelioma received this treatment. 156 patients (5%) were treated with PIPAC. In total 510 patients, (15%) received an operation that included a peritonectomy. For 293 patients (9%) both peritonectomy and HIPEC were noted simultaneously. The total distribution of patients for each treatment option can be observed in Fig. 1.

The mean length of hospital stay was 9 days, with a standard deviation of 11. However, there was a large variation recorded within this data, as can be observed in Table 1. The longest stay was recorded for HIPEC with 18 days on average, followed by patients with peritonectomy with 15 days. The shortest hospitalizations were observed after PIPAC with a mean of 3 days. Once again, the recorded standard deviations showed great variability in the data, as shown in Table 1.

**Table 1:** Average length of stay for each treatment option recorded during 2019 to 2024 ± the standard deviation. Data is given in days. “All Patients” classifies all recorded cases of peritoneal mesothelioma

Year	All Patients	HIPEC	Peritonectomy	PIPAC
2019	8 ± 10	22 ± 16	15 ± 16	4 ± 4
2020	9 ± 10	22 ± 15	15 ± 15	3 ± 2
2021	8 ± 10	20 ± 16	14 ± 13	3 ± 2
2022	9 ± 11	18 ± 12	15 ± 12	2 ± 1
2023	9 ± 14	19 ± 13	17 ± 17	2 ± 1
2024	10 ± 13	18 ± 12	15 ± 12	2 ± 1

Regarding gender distribution slightly more male patients were affected, with a mean of 58%. 52% of patients treated with HIPEC were male. For PIPAC 88% of recorded cases were performed on male patients. Patients that received peritonectomy were 56% male (Table 2).

**Table 2:** Gender Distribution. The data depicts how many patients diagnosed with peritoneal mesothelioma and receiving these treatment options were male. Information is given as the percentage of total patients treated with this procedure during the year. “All patients” refers to the total number of peritoneal mesotheliomas

Years	All Patients	HIPEC	Peritonectomy	PIPAC
2019	55	55	53	76
2020	62	59	62	90
2021	60	50	63	96
2022	60	62	62	100
2023	52	48	51	70
2024	57	36	47	97

**Fig. 2:**
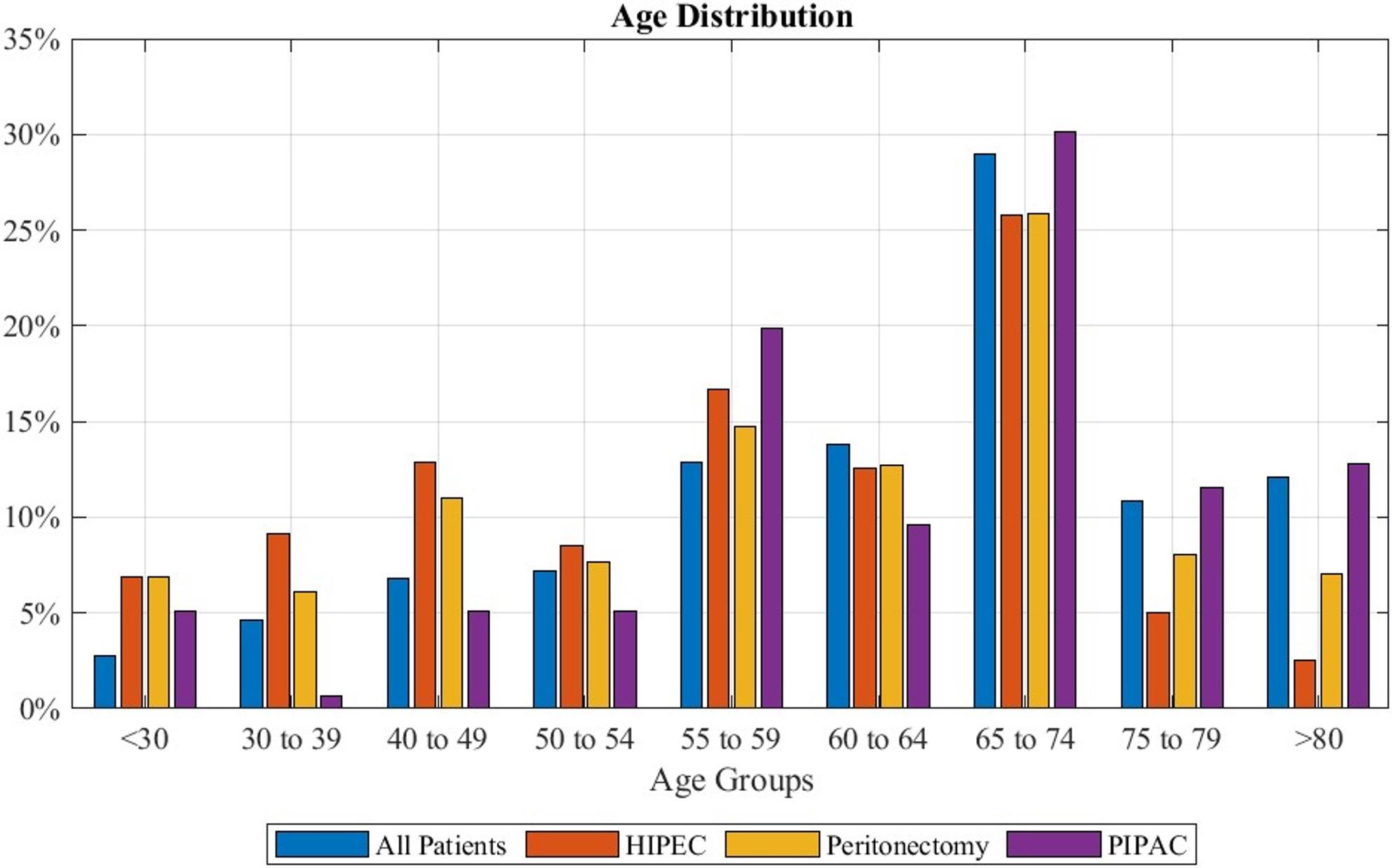
Age Distribution separated by performed treatment: “All patients” depicts overall patients diagnosed with peritoneal mesothelioma. Percentages refer to the total number of patients coded with the listed treatment option during the observed time frame of 2019 to 2024

The age range of patients can be observed in Fig. 2. The median age was 65 to 74, with between 23% and 35% of patients recorded in the years 2019–2024 being of this age. Patients undergoing HIPEC had a median age of 55 to 59 years. However, most patients were 65 to 74 years with 18% to 36% of patients falling in this age-range. Admits the patients recorded receiving PIPAC 18% to 52% were 65 to 74 years, marking this the median and peak age group. For those coded with peritonectomy a median of 60 to 64 years was calculated, with most patients (between 19% and 32%) being 65 to 74 years.

**Fig. 3:**
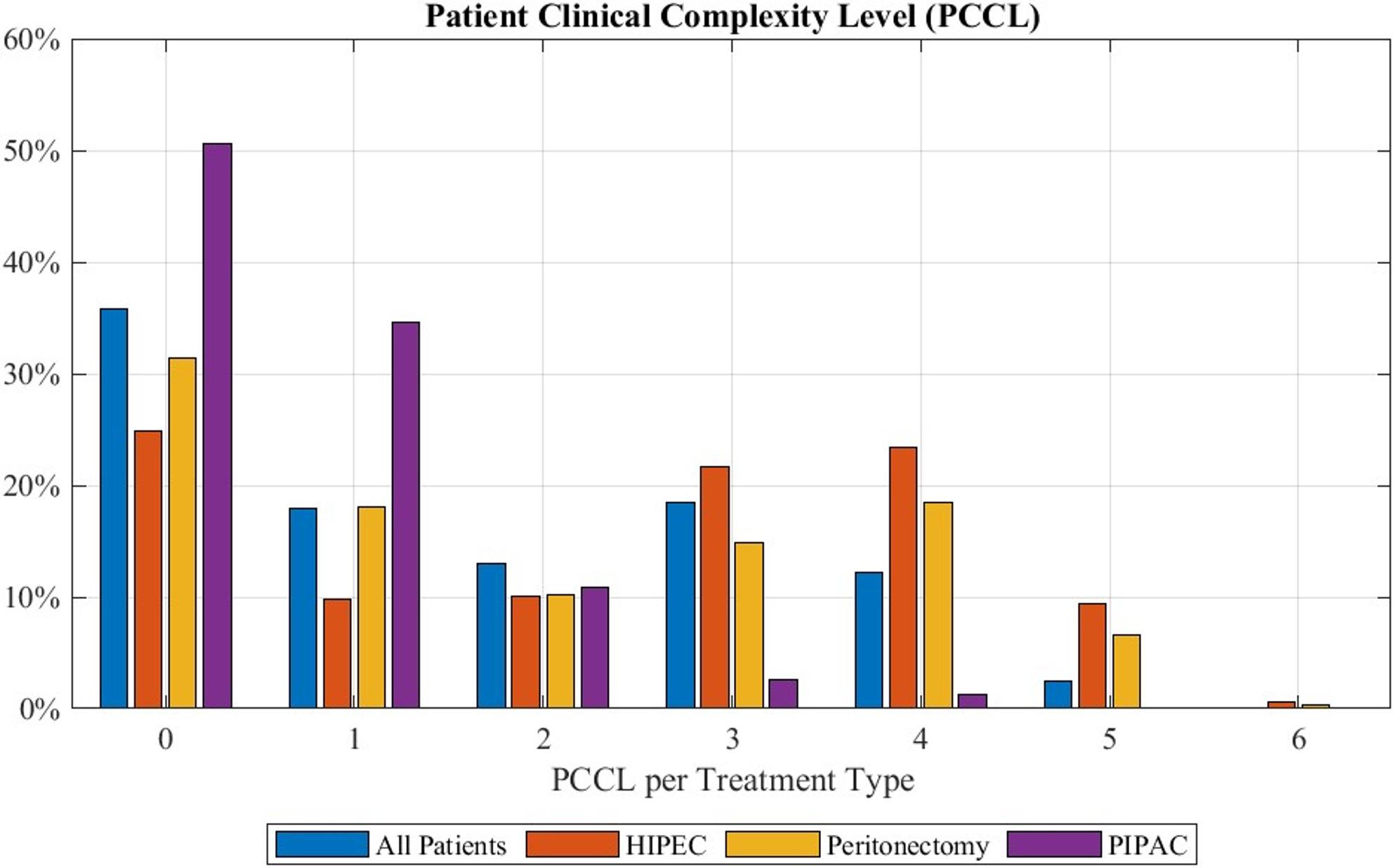
Patient Clinical Complexity Level for each treatment option: “All patients” gives the data of all patients diagnosed with peritoneal mesothelioma. Percentages refer to the total number of patients recorded with this treatment during the years 2019 to 2024

Regarding PCCL, as shown in Fig. 3, a median of 1 in patients with peritoneal mesothelioma was observed, while 32% − 43% of patients had a PCCL of 0, making this the mode. This indirectly indicates that most patients with this disease exhibited low to median complications. The performance of PIPAC exhibited a PCCL of 0 in 27% to 70% patients with this being also the median. Patients with HIPEC had a median PCCL of 3 with the majority of patients (12% to 43%) exhibiting a PCCL of 0. Peritonectomy resulted in a median PCCL of 2, however the mode was 0, as this was recorded for 17% to 46% of patients.

**Fig. 4:**
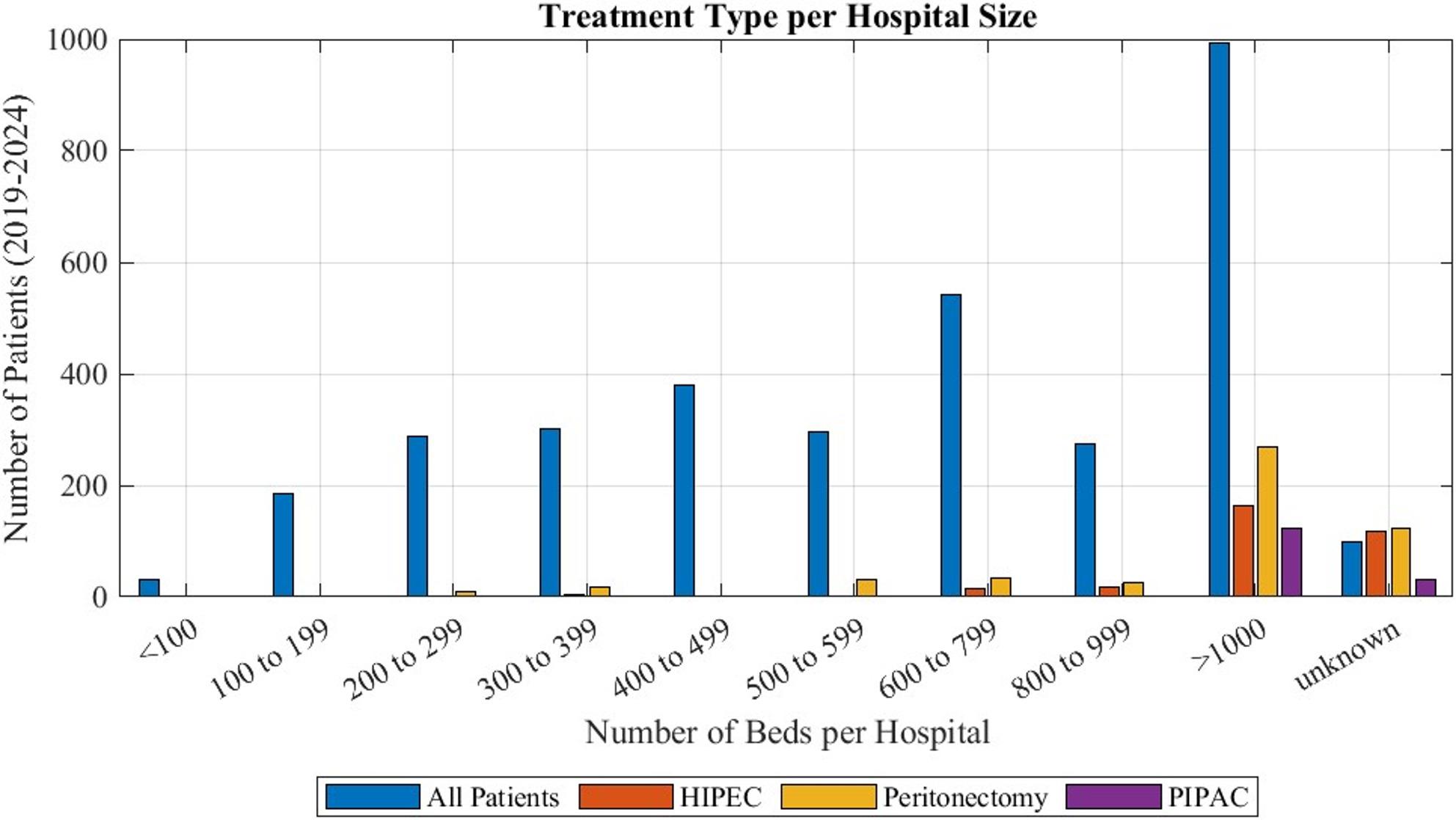
Hospital size at which the different procedures were performed during the years 2019 to 2024: Additionally, “All Patients” depict the hospital size at which all patients diagnosed with peritoneal mesothelioma were treated. “Unknown” marks data that was submitted without an assigned hospital size

Most patients with peritoneal mesothelioma (27% to 36% of them) were treated at maximum care hospitals with over 1000 beds, as can be seen in Fig. 4. Treatment of these patients was carried out at large, small and medium size hospitals. While some surgeries including peritonectomy were performed at hospitals as small as 200 beds, HIPEC was not recorded in hospitals with less than 600 beds. PIPAC was exclusively reported at maximum care hospitals with over 1000 beds. Some data was registered without information about the hospital size, so it had to be categorized as unknown. All treatment options were mostly carried out at hospitals with over 1000 beds (45% to 54% of patients for HIPEC, 67% to 100% for PIPAC, 43% to 58% for CRS).

**Fig. 5:**
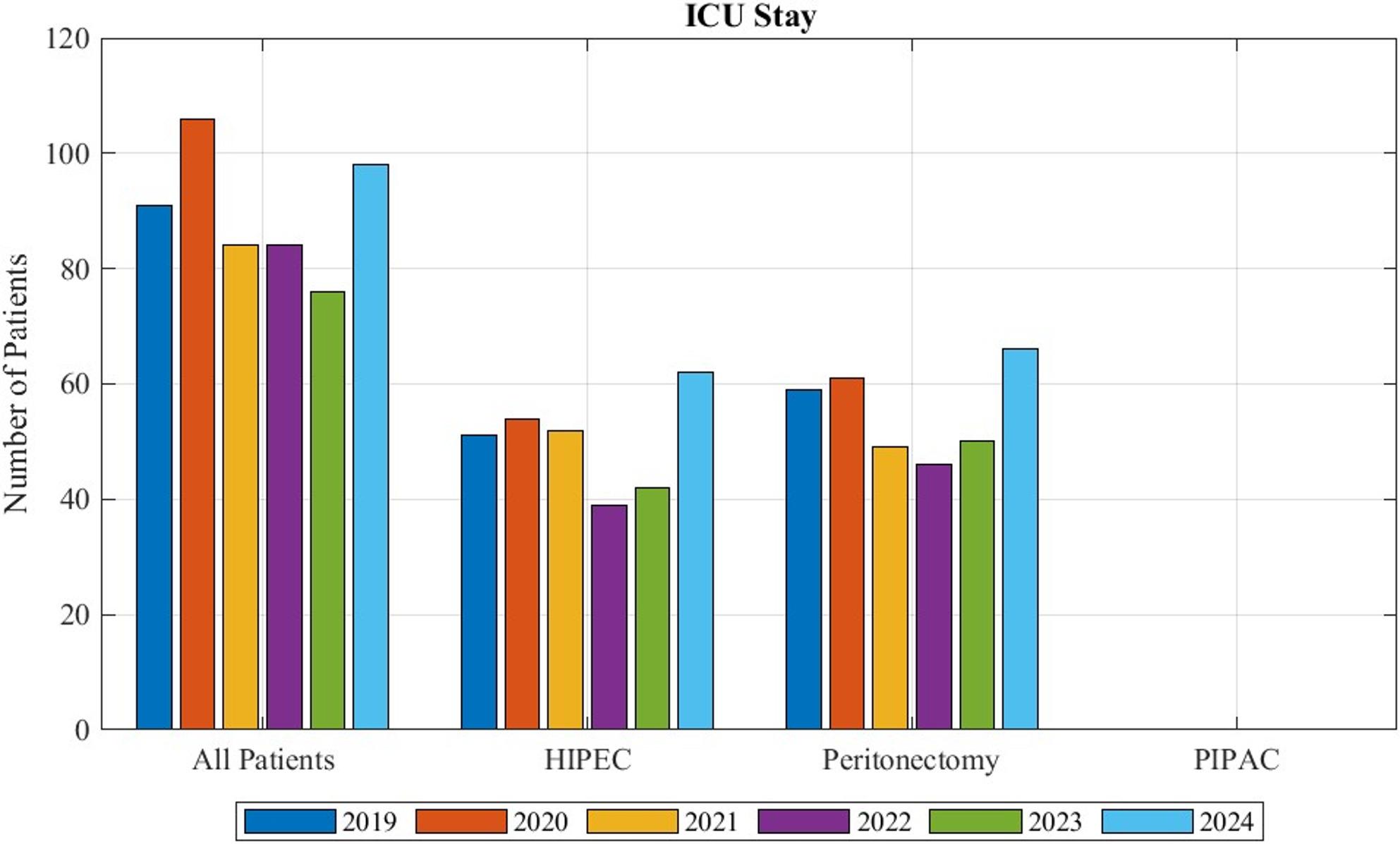
Patients with peritoneal mesothelioma in need of an ICU stay, separated by received treatments.” All patients” refers to the overall patients given the diagnosis

Figure 5 exhibits the number of patients with the diagnosis of peritoneal mesothelioma that had a stay at the intensive care unit. Overall, 16% of patients with peritoneal mesothelioma needed intensive care. 94% of patients receiving HIPEC had an ICU stay. For peritonectomy this was 65% of patients. During the examined time frame no ICU stays after the performance of PIPAC was recorded.

## Discussion

Our study gives a comprehensive overview of the epidemiology of peritoneal mesothelioma in Germany and examines the reality regarding the implementation of oncological guidelines. Additionally, with the examination of the PCCL and the ICU stays, insight into the complexities and complications associated with the current invasive treatment options is given.

With an average annual incidence of 0.68 per 100,000 inhabitants in Germany, our data reported a higher rate than previously published papers [2]. This highlights the importance of further research into the disease, because more patients are affected by it than previously estimated. We have also demonstrated that there is a significant decrease in incidence within the observed time frame. A possible explanation could the nationwide ban of asbestos in Germany and the resulting gradual disappearance of this compound from everyday life, as contact with it is known to influence the development of peritoneal mesothelioma [1, 5, 7]. However, it is unclear whether there are other explanations for this observation, especially with the ban taking effect in 1993.

We have demonstrated that in peritoneal mesothelioma surgical options are rarely applied. Assuming all patients coded with HIPEC also received peritonectomy and considering coding two procedures in one patient, only 16% of patients received a potentially curative treatment. With HIPEC being recorded in 9% of cases, PIPAC in 5%, peritonectomy in 15%, and with regards to dual coding, only 20% of all patients received any type of surgical therapy. The data show that despite CRS and HIPEC being the clearly recommended therapy by multiple guidelines [1, 7, 11], it is only performed in few cases. Since CRS and HIPEC have repeatedly been established to improve the overall survival of patients with borderline and malignant peritoneal mesothelioma [7, 14], the low application of this multimodal treatment is alarming, as it clearly demonstrates a shortcoming of the optimal treatment for these patients. Other European countries, such as Sweden, have recorded an increase in surgery [13]. France has established a nationwide network for rare peritoneal tumors, referred to as “Réseau National de prise en charge des Tumeurs Rares du Péritoine” (RENAPE), that connects experts from different specialties, such as surgery, oncology, or pathology [15, 16], ultimately providing patients with faster access to quality care [16]. Our data over this five-year period did not exhibit a similar trend for Germany. This leads to the conclusion that this must be based on the fact that German physicians rarely consider this type of treatment. The reasoning behind this observation could be diverse. In previous papers biphasic histology, Ki-67 > 9% and PCI > 17 have been considered contraindications for CRS and HIPEC [3]. However, in more recent times it is recommended to subject these patients to the operation after neoadjuvant treatment [1, 7]. It is possible, that a part of the low numbers of HIPEC performed in Germany could be due to physicians still operating by the outdated recommendations.

A possible explanation for the low numbers of potentially curative therapies could be the fear of complications arising from CRS and HIPEC [17]. As this is a highly invasive procedure, severe adverse effects such as infection, hematotoxicity, renal toxicity, pulmonary complications, and sever bowel injuries, like ileus and bowel perforation, as well as anastomosis insufficiency, can occur [5, 18, 19]. In our study the applied indicator for patient complexity, the PCCL-score, showed a higher score for patients undergoing this treatment than for those with the disease itself. This means, that patients receiving this treatment are recorded to have more additional diagnoses than patients without surgery, which indicates more complications. But the consequence of no adequate treatment is a significantly shorter survival [7, 14]. In fact, in comparison to other major oncological operations, that are well established and routinely performed, CRS and HIPEC have demonstrated rather low morbidity and mortality [17]. Therefore, the risk of surgery should not deter patient referral.

Another explanation could be that HIPEC - or surgery at all - was simply not considered due to physicians not being familiar with this treatment option. Previous research in the United States has demonstrated this as a factor for the low application of the procedure [17]. Interestingly, there are currently no designated ESMO guidelines for peritoneal mesothelioma, which could be an indication of a lack of awareness for this disease. Of course, it could also be assumed that due to the broad accessibility of systemic therapy in Germany and limited options of surgical centers, patients with peritoneal mesothelioma are often referred to and treated by clinical oncologists. They naturally can lack insight into surgical possibilities. Additionally, there are financial incentives for oncologists to guide the patient to non-surgical treatments, as the DRG-system offers a better reimbursement for the systemic treatment than for the referral. This would demand subsequent action by changes in education and information of physicians and the implication of clear guidelines to alter the results observed in our study.

Naturally, the detected low rates of HIPEC application could simply be the result of patients not meeting the criteria to be candidates for CRS and HIPEC, as it has been shown by other investigators [5]. Meaning they could have an unfavorable histology, such as the sarcomatoid subtype, or simply present with an ECOG $\:\ge\:$ 2, lacking the physical fitness needed to endure this procedure [7]. Or there simply could be too much of a disease burden present at diagnosis to deem the patient resectable. This would include the patient presenting with metastasis outside of the abdomen, though in selected cases with small disease burden an operation could still be deemed feasible [7], tumor infiltration into the essential vascularization of the bowel or liver, or extensive small bowel invasion [14]. This would underline the importance of better diagnostic tools and identification of risk factors, leading to the development of screening programs.

However, this assumption seems not to be the major contribution to the observed effects. Assuming that many patients would be unfit for CRS and HIPEC treatment, the majority of these patients would likely still be eligible for PIPAC, considering that there are only few contraindications to this intervention [18, 19]. Nevertheless, our research showed that just like the other surgical options, PIPAC was only performed in a small number of cases. Even though, high-level evidence demonstrating a significant overall survival benefit from PIPAC is still missing, this intervention has been demonstrated as safe with promising oncological outcomes and potential benefit in selected patients [18]. It could even be used as a neoadjuvant treatment option [8]. In accordance with the previously published consensus [19] our data underlined the treatment safety with the median and mode PCCL of 0 indicating that most patients receiving this treatment experienced only minor complications. This is further supported by our finding that no patient with this treatment needed an ICU stay during the observed period. Thus, there should be no safety concerns deterring physicians from subjecting patients to PIPAC. Other causes of the low incidence of PIPAC could be a lack of awareness regarding not only the existence and indication of this procedure, but also of the expertise of centers performing it, hindering patient referral. Another factor is the economic aspect of the procedures. With the current German hospital reimbursement system, institutions make financial losses if they perform CRS and HIPEC for each patient, since the offered reimbursement usually does not cover the cost of the procedure [20]. Evidently, this creates an incentive for the hospitals to not offer the operation, making it harder for patients to receive proper care.

Interestingly, a discrepancy between HIPEC and peritonectomy could be recorded. Most of the time when HIPEC was coded, a peritonectomy was also coded for the same patient. However, for 246 patients receiving peritonectomy no HIPEC was reported. As there are recommendations not to perform intraperitoneal chemotherapy if the achieved cytoreductive score (CC-score) is greater than 1 [9], these cases could be an indicator for unsuccessful surgeries. Additionally, in 25 cases HIPEC was coded, but no peritonectomy was noted for the same patient. Because there are currently no indications for HIPEC without CRS, we assume this to be primarily an error in coding, with hospitals either, only reporting one of the procedures performed, or utilizing other codes to indicate CRS. Another possibility would be patients receiving CRS in other countries and being referred to Germany solely for the completion with HIPEC.

Another important aspect that can be derived from current literature is that peritoneal mesothelioma should always be treated at specialized centers [5, 15]. As the disease is rare and the operative techniques applied in CRS are of greater difficulty, requiring broad experience [11], treating the patients only at specialized centers allows for more experienced surgeons. This can improve the ability to reach the maximum level of cytoreduction [2, 21]. Regarding the observed discrepancy between the rates of surgery and HIPEC, there is more emphasis on the importance of specialized centers, as only at these levels of expertise for optimum surgery can be ensured. Additionally, it has been shown by Moran et al., that with greater experience of surgeons and centers, a decrease in complications and an increase in survival can be achieved [21]. There is also concern regarding resource allocation. For the performance of HIPEC and PIPAC, specialized equipment and staff training are required. The financial investment in offering these invasive treatments is justified only if enough patients can be acquired. All these factors could attribute to our observation that no HIPEC was applied in hospitals with less than 600 beds, despite surgeries being conducted in smaller hospitals. Interestingly, with PIPAC needing less resources and technical skills, we only recorded patients treated at maximum care hospitals receiving this treatment. Consequently, financial reasoning cannot be the only explanation behind our observations. One possible influence could be the structure of the German healthcare system, in which larger hospitals are designated as centers for highly specialized medicine and research. This could encourage these hospitals to be more aware of new techniques and changes in guidelines and implement them faster into their standard of care. Small and medium-sized hospitals are designated to provide emergency relief and basic care. This attribution is well-known in the German public, as well as among referring physicians, which could result in more patient-referral to larger hospitals for these specialized treatments of this rare disease.

All these findings and possible explanations support the importance of the implantation of a centralized organization and registry to better navigate the care of patients with peritoneal mesothelioma and other rare diseases in Germany.

Naturally, there are limitations to the presented study. For example, only data marked with the utilized ICD-10 code and OPS-codes could be identified. Therefore, differently coded procedures and assigned diagnoses could not be detected, which could alter our results. For instance, we opted to identify surgery by the performance of peritonectomy, as it is an essential part of CRS in peritoneal mesothelioma [2, 10], regardless of the infiltration of other organs. However, other codes could have been assigned to the performed operations. We decided to accept this loss of information, because CRS can be a surgery with multiple different components and therefore there is a vast variety of different possible codes, that do not necessarily indicate CRS. For example, an operation could have been classified under the term “debulking surgery,” which does not note a specific OPS-code and is only incorporated within the code for “resection of tissue in the abdominal region without certain organ assignment.” Including identifiers like these would lead to unspecific data, as they could contain any type of surgery and need not be an indication of CRS. Also, it is possible that some hospitals could have used other OPS-codes for HIPEC or PIPAC, that also indicate an application of chemotherapy. Especially since the specific code for PIPAC was introduced in the 2019 OPS-catalogue and therefore may not have been familiar to some centers during the first examined years. Since there are many OPS-codes of varying degrees of specificity regarding chemotherapy the degree of alternatively coded data could not be identified. For the same reason we opted to not filter codes of systemic chemotherapy, as the sheer number and variety of those would have resulted in unspecific data with a low informative value. Also, there is no ICD-10 code differentiating between the histological subtypes of peritoneal mesothelioma, despite there being significant discrepancies in the recommended treatment [2]. Therefore, our data could be altered by a high number of sarcomatoid subtypes, which are not eligible for surgery [2]. Though with the occurrence of the sarcomatoid subtype being reported as rare [5, 22], confounding arising from this consideration can be assumed as limited. Another limitation concerns the data for the year 2024. At the time of publication, for this time frame, unlike for the other time frames, there is no retrospective accumulation of information. This could mean that some reports might still be missing, causing an alteration of our findings.

## Conclusion

Peritoneal mesothelioma constitutes a challenging disease that requires specialized treatment with CRS and HIPEC [1, 7, 11]. Patients that do not meet the criteria for the primary treatment, should be treated with PIPAC or systemic chemotherapy [2]. Our research gives important epidemiological information about this disease in Germany and highlights a grave discrepancy between the guideline recommendations and actual patient treatment. Additionally, this work underlines the importance of treatment of peritoneal mesothelioma in specialized centers. Future research is necessary to identify the reason behind the observed discrepancies and to develop measures to counteract them to provide optimal patient care and maximized overall survival.

## Data Availability

The datasets supporting the conclusions of this article are available under the “InEK DatenBrowser”. Due to privacy policy the databank is not publicly accessible, but for the purpose of medical research and upon permission from InEK (https://www.g-drg.de) access can be granted.
